# Integrating single-cell RNA-sequencing and bulk RNA-sequencing data to explore the role of mitophagy-related genes in prostate cancer

**DOI:** 10.1016/j.heliyon.2024.e30766

**Published:** 2024-05-09

**Authors:** Zong-Yan Liu, Ruo-Hui Huang

**Affiliations:** a Department of Pharmacy, Ganzhou People's Hospital (Ganzhou Hospital-Nanfang Hospital, Southern Medical University), Ganzhou, Jiangxi, 341000, China; b Department of Urology, First Affiliated Hospital of Gannan Medical University, Gan Zhou, Jiang xi, 341000, China; c Jiangxi Stone Prevention Engineering Technology Research Center, Gan Zhou, Jiang xi, 341000, China

**Keywords:** Prostate cancer, scRNA-seq, RNA-seq, Biomarkers, Mitophagy-related genes, Prognosis model

## Abstract

Prostate cancer (PCa) is the most common malignancy of the male urinary system. Mitophagy, as a type of autophagy, can remove damaged mitochondria in cells. Mitophagy-related genes (MRGs) have been shown to play critical roles in the development of PCa. To this end, based on the comprehensive analysis of RNA-seq and scRNA-seq data of PCa samples and their controls, this paper identified PCa subtypes and constructed a prognostic model. In this paper, we downloaded scRNA-seq and RNA-seq data from Gene Expression Omnibus (GEO) and TCGA database. Based on the R package “Seurat” to process the scRNA-seq data, a total of five cell types were identified. Each cell population was scored based on the R package “AUCell” and using the intersection genes between MRGs and each cell population. The B cell population was then identified as a high-scoring cell population. Differentially expressed genes in RNA-seq data were identified based on the R package “limma” and intersected with previously intersected genes. Then, based on univariate Cox regression analysis and Lasso-Cox regression analysis, the prognostic genes were screened, and the risk model was constructed (composed of ADH5, CAT, BCAT2, DCXR, OGT, and FUS). The model is validated on internal and external test sets. Independent prognostic analysis identified age, N stage, and risk score as independent prognostic factors. This paper's risk models and prognostic genes can provide a reference for developing novel therapeutic targets for PCa.

## 1 Introduction

Prostate cancer (PCa) mainly occurs in older men. As one of the most common malignancies of the urological system, PCa is the second leading cause of cancer death in American men [1]. In the global male cancer-related mortality, PCa ranks fifth [2]. Due to population growth and aging, it is estimated to rise to nearly 2.3 million new cases and 740,000 deaths by 2040 [3]. Therefore, an in-depth exploration of the molecular mechanism in the development of PCa is of positive significance for early diagnosis and the development of new therapeutic methods [4].

Mitophagy is a specialized form of autophagy designed to clear damaged, defective, dysfunctional mitochondria [5]. Recent studies have shown that genes such as PINK1 and BNIP3L are closely related to this process [6,7]. Mitophagy has also been shown to play a role in the progression of various cancers, including PCa. For example, Han et al. found that Abiraterone and MDV3100 inhibited the proliferation and promoted apoptosis of PCa cells through mitophagy [8]. Surbhi Chouhan and colleagues' research revealed TNK2-mediated mitochondrial energy regulation as a vulnerability of PCa cells, and provided evidence for TNK2 inhibitors as potential therapeutic strategies inducing cancer-specific mitochondrial autophagy [9].

The key molecular mechanism of mitophagy-related genes (MRGs) in the occurrence and development of PCa is still unclear. This paper explored the expression landscape and scoring of MRGs in different cell clusters based on comprehensive bioinformatics analysis. Prognostic MRGs were screened based on RNA-seq data and used to identify PCa subtypes and construct prognostic models. Our findings will provide potential prognostic biomarkers and therapeutic targets for PCa.

## 2 Method

### 2.1 Acquisition of raw data

In this paper, scRNA-seq data of three PCa samples were obtained from the GSE153892 dataset of the GEO database (https://www.ncbi.nlm.nih.gov/geo/). We searched for “mitophagy” in the genecard database (https://www.genecards.org/) and obtained 1000 mitophagy related genes (MRGs). Then, 137 MRGs were obtained from previous literature [10]. By merging two sets of MRGs, 1035 MRGs were obtained for subsequent analysis. For transcriptome data, we first downloaded 52 control samples and 501 PCa samples from the TCGA-PRAD cohort from the TCGA database (https://portal.gdc.cancer.gov/). 179 PCa samples were obtained from the GSE70770 dataset of the GEO database. Samples from the TCGA-PRAD cohort were randomly divided into a training set (52 control samples and 501 PCa samples) and an internal test set (52 control samples and 501 PCa samples). Samples from the GSE70770 dataset serve as an external test set.

### 2.2 Processing and analysis of scRNA-seq data

This paper converts 10X scRNA-seq data into Seurat objects via the Seurat package [11]—screen for genes expressed in at least three cells and cells with 300 genes detected during transformation. The quality control indicators need to meet the ratio of mitochondrial genes greater than 15 %, the number of genes contained in cells is more significant than 6000 or less than 100, and the number of reads of each gene in each cell is more significant than 20,000. Then use the “FindVariableFeatures” function to get the top 2000 hypervariable genes. Dimensionality reduction and clustering are based on PCA and Uniform Manifold Approximation and Projection (UMAP). Based on the “singleR” software package, different cell clusters were annotated with cell type [12]. Based on the “FindAllMarkers” function, the differentially expressed genes (DEGs) satisfying the absolute value of logFC greater than 0.25 and p<0.05 among different types of cells were identified. Take the intersection of DEGs and MRGs. Cell clusters were scored based on intersecting genes using the AUCell software package. Gene Ontology (GO) enrichment and metascape analysis were performed on the DEGs of high-scoring cell populations based on the ClusterProfiler software package and the metascape database (https://metascape.org), respectively. Finally, the CellChat software package [13] was used for intercellular communication analysis and network visualization.

### 2.3 Differential expression analysis and mutation analysis

In this article, the R package “limma” was used to deduplicate genes in the expression matrix of the TCGA-PRAD queue, and gene expression data with expression levels greater than 0.1 were retained. Next, perform differential analysis on the data after quality control to identify genes with differential expression between normal tissues and Pca tissues. FDR was used for p-value testing. |Log2FC |>0.5 and FDR<0.05 were used as screening criteria for differentially expressed genes (DEGs), and DEGs were used for the next analysis. In addition, we downloaded copy number variation (CNV) data for the TCGA-PRAD queue from the UCSC Xena (http://xena.ucsc.edu/) database. The gain and loss plots of CNV were plotted using the barplot (.) function.

### 2.4 NMF clustering algorithm

In this paper, a clustering study was performed based on the expression of the intersection genes in the training set data of the TCGA-PRAD dataset. Specifically, clustering based on the non-negative matrix factorization (NMF) algorithm was implemented using the R package “NMF” [14]. The number of clusters was set to 2-10, and 100 iteration cycles were performed. The most suitable number of clusters was determined according to the discrimination of each subtype under different cluster numbers.

### 2.5 Construction and verification of prognostic model

Univariate Cox regression analysis was first performed on the training set to identify prognostic-associated intersection genes. Genes with p<0.05 were entered into regression analysis of least absolute shrinkage and selection operator (LASSO). In this paper, after implementing LASSO based on the “glmnet” software package, the memory-unscreened genes were input into the multivariate Cox regression analysis algorithm. The patient's risk score in the prognostic model was calculated according to the following formula.

$$\text{riskscore}=\sum_{i=1}^{n}\;\text{coef}\;\left(i\right)*\text{expr}\;\left(i\right)$$

coef(i), expr(i), and n represent the coefficient, expression value, and number corresponding to the genes used to build the model, respectively. We divided all diseased samples into high and low-risk groups based on the median value of each patient's risk score. To confirm the predictive ability of the model, this paper evaluated the high and low survival curves based on Kaplan-Meier survival curve analysis (implemented by survminer and ggrisk software packages), time-dependent receiver operating characteristic (ROC) curve analysis (implemented by timeROC software package). Survival from recurrence (BCR) in risk groups. The most significantly enriched pathways between high and low-risk group samples were identified by gene set enrichment analysis (GSEA). Pathways with significantly different enrichment scores between the two risk groups were determined by gene set variation analysis (GSVA). The gene sets used during the analysis of GSEA and GSVA are c5.go.v7.4.symbols.gmt and c2.cp.kegg.v7.4.symbols.gmt, respectively. In addition, based on the human protein mapping HPA database (https://www.proteinatlas.org/) to explore the prognosis related gene expression in normal and cancerous tissue.

### 2.6 Independent prognostic analysis based on the nomogram model

To confirm that the nomogram model can be used as an independent prognostic factor, this paper constructed a nomogram model based on risk score and clinical characteristics (age, N stage, and T stage).

### 2.7 Immune-related analysis

In this paper, based on the ssGSEA algorithm, the immune infiltration analysis was performed on the diseased samples of the TCGA-PRAD cohort, and the infiltration abundance of various immune cells in the samples and the immune activity scores of various immune functions in the samples were evaluated. In addition, the possibility of tumor immune escape was assessed based on the gene expression profiles of PCa samples and through the TIDE database (http://tide.dfci.harvard.edu/).

### 2.8 Statistical analysis

All statistical analyses involved in this article were performed using R software version 4.2.2. All P values are two-sided, and statistical significance is P<0.05.

## 3 Result

### 3.1 Results of scRNA-Seq analysis

**Fig. 1:**
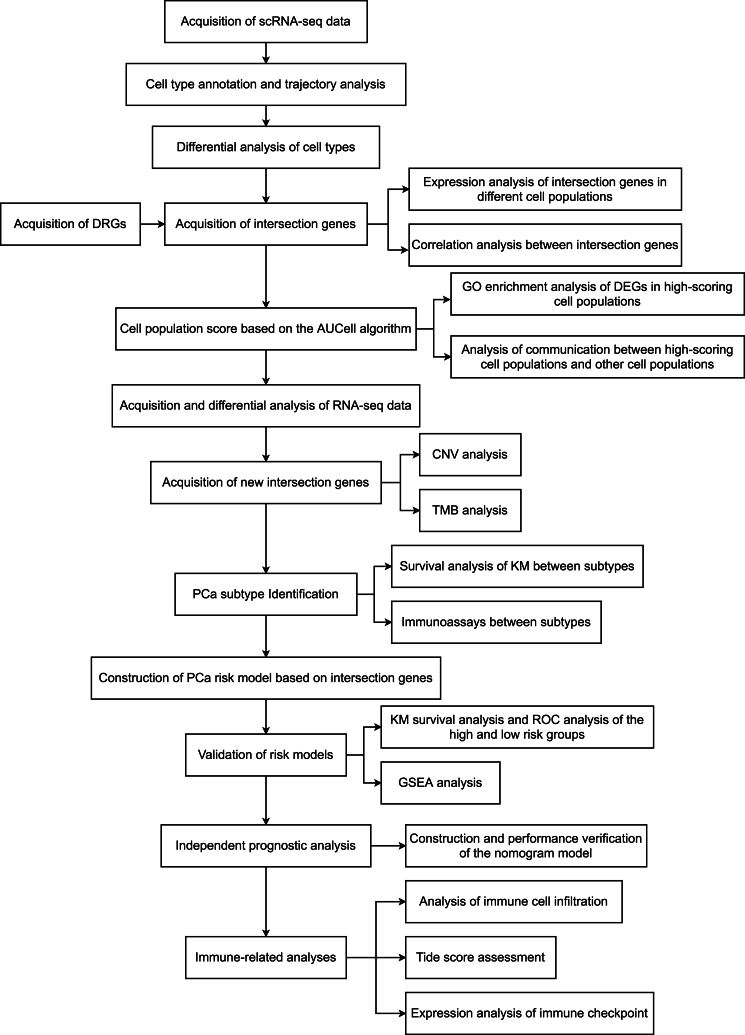
Overall flow chart of the paper.

**Fig. 2:**
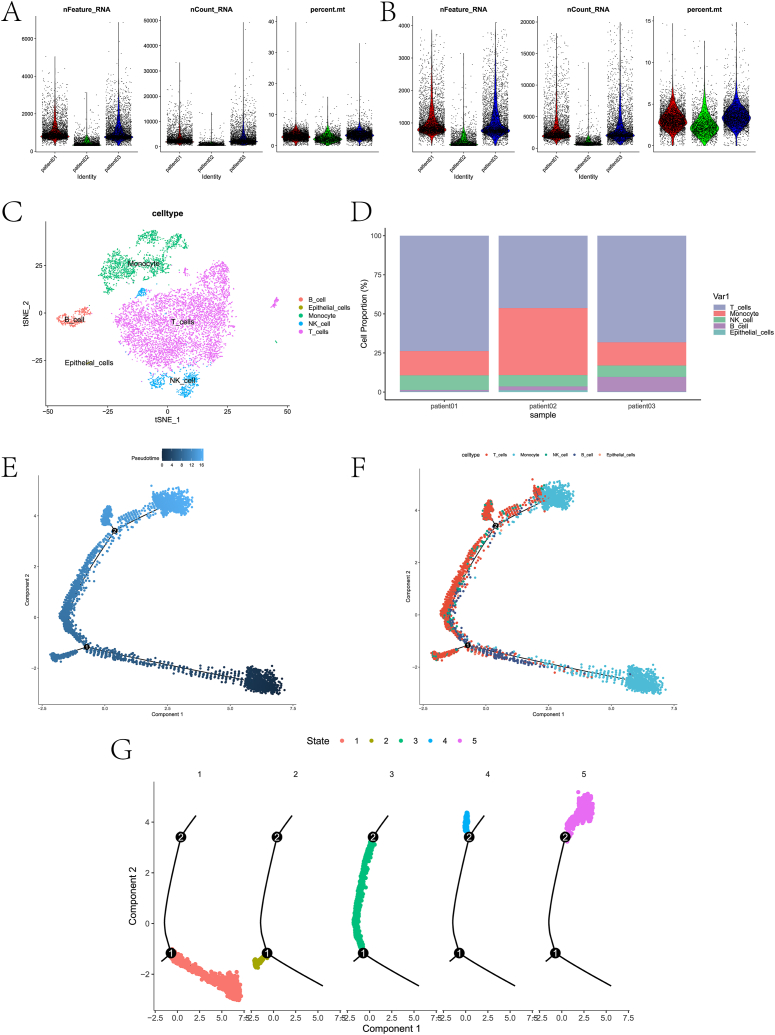
Pretreatment and type identification of RNA-SEQ data. A and B are violin charts of nFeature, nCount and mitochondrial gene proportion before and after quality control, respectively. C is the result of identification of different cell types. D is a histogram of the proportion of different cell types in the sample. E-G is the result of different types of cell trajectory analysis, pseudo-time analysis and state analysis respectively.

**Fig. 3:**
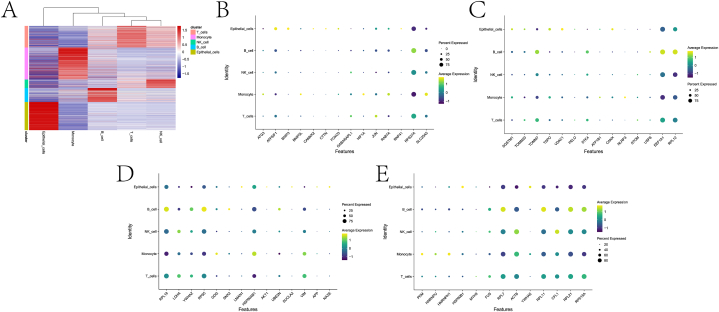
Analysis of differences among cell populations and expression of intersection genes. A is the heat map of DEGs expression in each cell population. B-E shows the expression of intersection genes between different cell populations.

Fig. 1 shows the technical roadmap of this paper. First, the scRNA-seq data of the GSE153892 dataset was analyzed. Qualified cells were retained after quality control (Fig. 2A–B). After hypervariable gene identification, PCA, and TSNE analysis, different cell clusters were annotated based on the “singleR” software package (Fig. 2C), and five cell types (B cells, epithelial cells, monocytes, NK cells, and T cells) were identified. We also counted the proportions of the five cell types in the three samples (Fig. 2D). Among them, the patient02 contains more monocytes. Trajectory analysis of different types of cell populations based on the Monocle software package. We observe that monocytes correspond to states 1 and 5. T cells were present in all states (Fig. 2E–G). In this paper, different types of cells were analyzed differentially. Fig. 3A presents the expression heatmap of the top-ranked DEGs in each cell cluster. After intersecting all DEGs and MRGs (see the MRG.txt file in the supplementary material for details), 56 intersection genes were obtained. We explored the expression of these genes in different cell types (Fig. 3B–E).

**Fig. 4:**
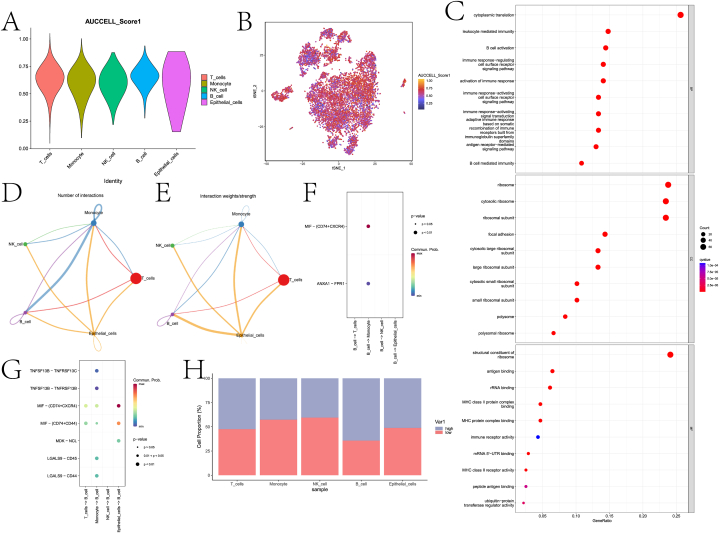
Correlation analysis of AUCell and cell communication. A is a violin diagram based on AUCell's scores of different cell populations. B gives AUCell scores of different cell populations. C is the result of GO enrichment of B cell group DEGs. D and E are network maps of the number and weight of interactions between different cell populations, respectively. F and G are the signaling pathways that use B cells as receptors and ligands to communicate with other cells. H is a histogram of the proportion of high and low scoring cells in different cell types.

It was further based on the AUCell algorithm and using intersection genes to score different types of cell populations (Fig. 4A–B). After dividing all cells into high and low-scoring cells according to the median score, we counted the proportion of high and low-scoring cells in different types of cells (Fig. 4H). The B cell population received the highest score. GO enrichment analysis was performed on DEGs of the B cell population. The Discussion section will explore the relationship of related pathways to PCa. We also explored the number and strength of interactions between different cell populations by cell communication analysis (Fig. 4D–E). In addition, we used B cell populations as receptors and ligands, respectively, to explore the pathways involved in their communication with other types of cell populations (Fig. 4F–G). The MIF pathway plays a crucial role in communication networks.

### 3.2 DEGs identification and related analysis

**Fig. 5:**
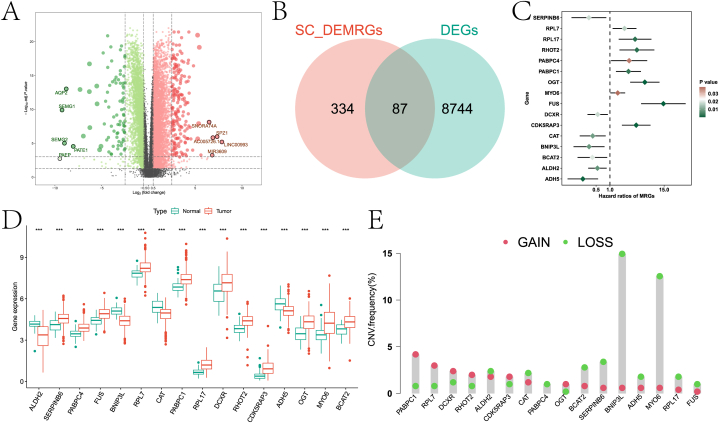
Analysis of RNA-seq data. A is the volcano map obtained from the difference analysis of the TCGA-PRAD cohort training set data. B is the Wayne diagram obtained by intersection of differential mitochondrial autophagy genes in DEGs and single-cell sequencing analysis. C is the forest map obtained by single-factor Cox regression of intersecting genes. D is a boxplot reflecting the expression of intersection genes in the control group and the diseased group. E is the CNV analysis result of intersection gene.

In this paper, we first performed differential expression analysis on the RNA-seq data in the training set of the TCGA-PRAD cohort. Fig. 5A shows the volcano plot of the differential analysis, where the marked genes are the top significantly upregulated and downregulated. We further intersected it with the intersection genes obtained in scRNA-seq data analysis, and obtained a total of 87 intersection genes (Fig. 5B). By univariate Cox regression analysis, 16 genes that were significantly associated with the prognosis of PCa patients were retained (Fig. 5C). There were significant differences (p<0.01) in the expression levels of these genes between the control group and the diseased group (Fig. 5D). In addition, we identified changes in regulators with CNV signature on chromosomes (Fig. 5E).

### 3.3 PCa subtype identification based on intersection genes

**Fig. 6:**
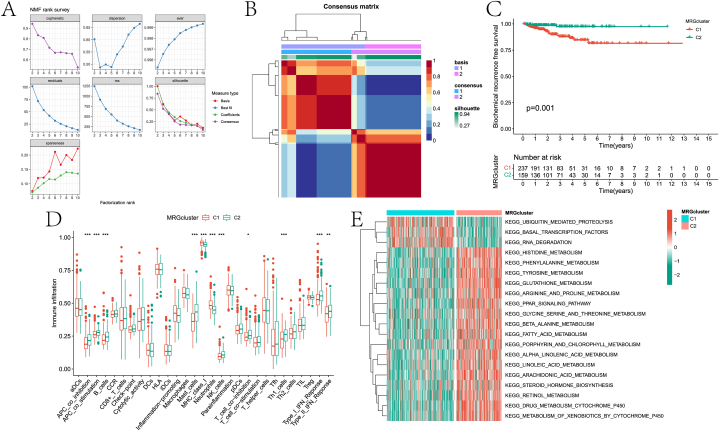
Subtype identification results of PCa. A is the factorization rank of the NMF algorithm with k values from 2 to 10. B is the consensus matrix heat map when k is 2. C is the result of KM survival analysis for two subtypes. D is the boxplot of the difference analysis of immune cells between different subtypes. E is the GSVA analysis result of two subtypes involved in the pathway.

Here, we identified the PCa subtype based on the expression of prognostic genes identified by univariate Cox regression analysis. Specifically, this paper performs clustering based on the NMF algorithm. The cophenetic correlation coefficient was used to determine the optimal number of clusters, and after comprehensive consideration, k = 2 was selected as the optimal number of clusters (Fig. 6A). It can be seen from Fig. 6B that the two subtypes have clear boundaries, indicating that reliable clustering results can be obtained under this parameter. Significant differences in BCR between the two subtypes were found by KM survival analysis (Fig. 6C). Differences in the abundance of immune cell infiltration and immune function scores in different subtype samples were assessed by the ssGSEA algorithm (Fig. 6D). Antigen-presenting cell co-inhibition (APC_co_inhibition), antigen-presenting cell co-stimulation (APC_co_stimulation), B cells, mast cells, major histocompatibility complex class I molecules (MHC_class_I), Neutrophils, NK_cells, Th1 helper cells (Th1_cells) and I The infiltration abundance/function scores of Type_I_IFN_Reponse in samples of different subtypes were significantly different. Furthermore, the differences in pathway scores involved in the two subtypes were assessed by GSVA analysis (Fig. 6E).

### 3.4 Construction and verification of risk model based on intersection genes

**Fig. 7:**
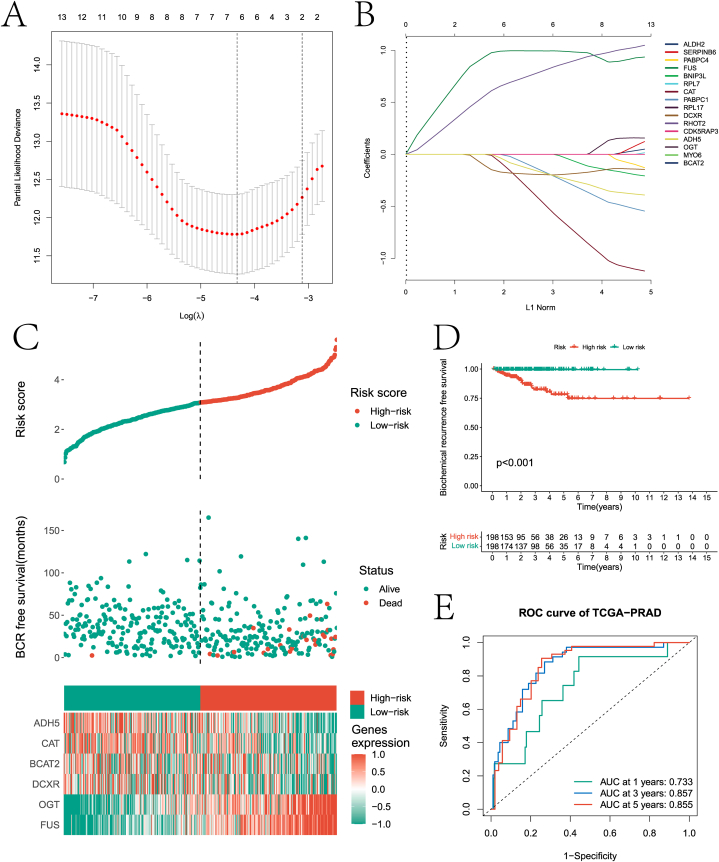
Risk model construction of Pca. A and B are processes based on the LASSO algorithm to screen genes used to build risk models. C presents risk curves, scatter plots of risk factors and heat maps of prognostic genes used to construct risk models based on internal test set data. D is the KM survival analysis results of high and low risk groups. E is the ROC curve that predicts 1 -, 3 -, and 5-year survival of patients in both groups based on the risk model.

**Fig. 8:**
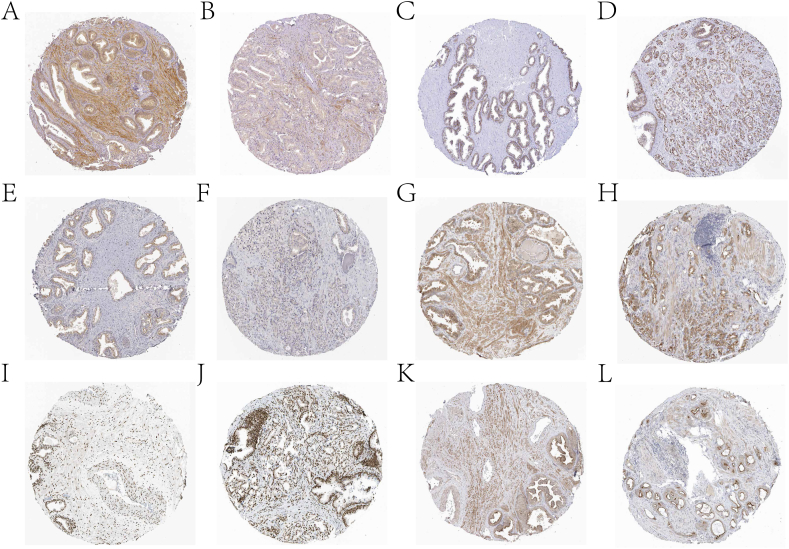
Immunohistochemical staining showed the expression of six genes at the protein level. A, C, E, G, I and K are immunohistochemical staining images of ADH5, BCAT2, CAT, DCXR, FUS and OGT in normal tissues, respectively. B, D, F, H, J and L are the immunohistochemical staining images of ADH5, BCAT2, CAT, DCXR, FUS and OGT in PCa tissues, respectively.

**Fig. 9:**
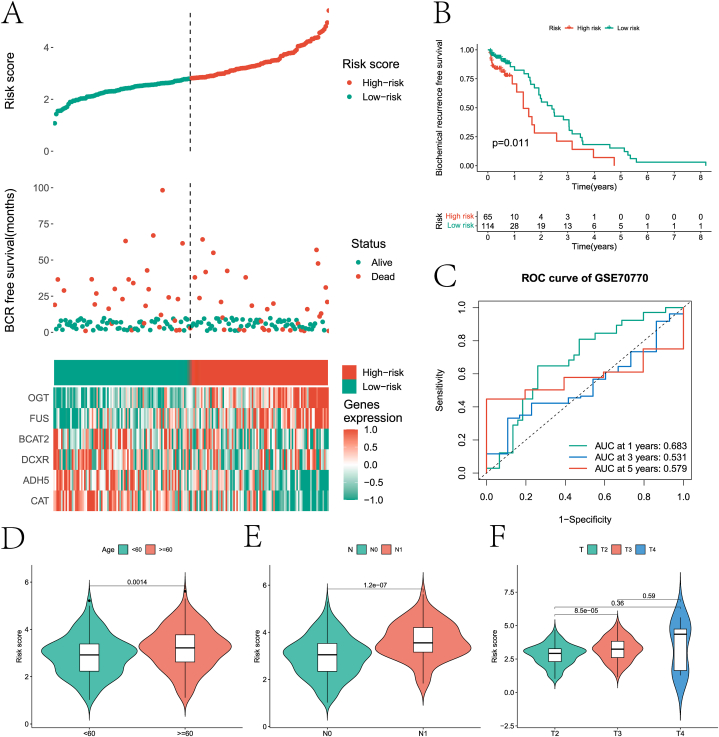
Validation of risk models. A is a risk curve based on an external test set, a scatter plot of risk factors, and an expression heat map of prognostic genes used to construct the risk model. D is the KM survival curve of high and low risk groups based on external tests. E is the ROC curve that predicts 1 -, 3 -, and 5-year survival of the two groups of patients in the external test based on the risk model. D-F was the difference in age, N stage and T stage between the two groups.

In this paper, LASSO regression analysis was performed on the 16 prognostic genes screened by the previous univariate Cox regression analysis to reduce the number of genes in the final risk model (Fig. 7A–B). Six genes (ADH5, CAT, BCAT2, DCXR, OGT, and FUS) were retained after screening. Then a diagnostic model was constructed by multivariate Cox regression analysis. All PCa patients were divided into high and low risk groups according to the risk score provided by the diagnostic model for each PCa sample. Fig. 7C demonstrates the rationality and reliability of the risk model construction through the risk curve of the internal test set, the scatter plot of the sample risk score, and the expression heat map of the 6 genes in the two groups. Through KM survival curve analysis, it was found that there was a significant difference in the BFS of the internal test set between the two groups of samples (Fig. 7D). The ability of the risk model in predicting 1-, 3-, and 5-year survival of PCa patients on the internal test set was evaluated by ROC curves (Fig. 7E). Finally, the potential value of the six genes was verified by the HPA database. The results in Fig. 8A-L show that six genes have significant differential expression levels in PCa and control samples. In addition, this paper further validates the model based on the external test data of the GEO database (Fig. 9A–C). In addition, we explored the differences in clinical factors (age, T stage, and N stage) between high- and low-risk group samples (Fig. 9D–F).

### 3.5 Independent prognosis analysis

**Fig. 10:**
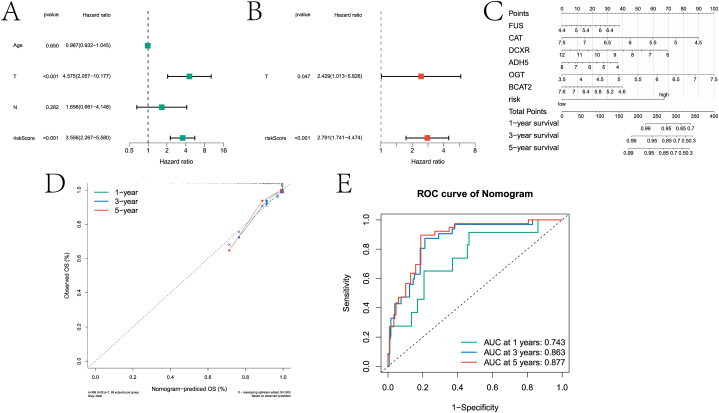
Independent prognostic analysis results. A and B were the results of univariate and multivariate Cox regression analysis of clinical factors and risk scores, respectively. C is a nomogram model based on prognostic genes and risk scores. D is the calibration curve of the nomogram model. F is the ROC curve that predicts 1 -, 3 -, and 5-year survival of patients based on a nomogram model.

Furthermore, based on the risk score and clinical factors (age, T stage, and N stage), this paper screened the independent prognostic factors of PCa and constructed a nomogram model. Specifically, this paper screened T stage and risk score as independent prognostic factors based on univariate and multivariate Cox regression analysis (Fig. 10A–B). Nomogram models were constructed based on two independent prognostic factors and evaluated against calibration curves (Fig. 10C–D). Likewise, we considered the ability of the nomogram model to predict patient 1-, 3-, and 5-year survival based on ROC analysis (Fig. 10E). The results showed that the ROC of the model to predict the 1-year, 3-year, and 5-year survival rate of patients reached 0.743, 0.863 and 0.877, respectively. It is confirmed that the model's predictive ability is better than the risk model's.

### 3.6 Enrichment analysis and immune landscape of high and low risk groups

**Fig. 11:**
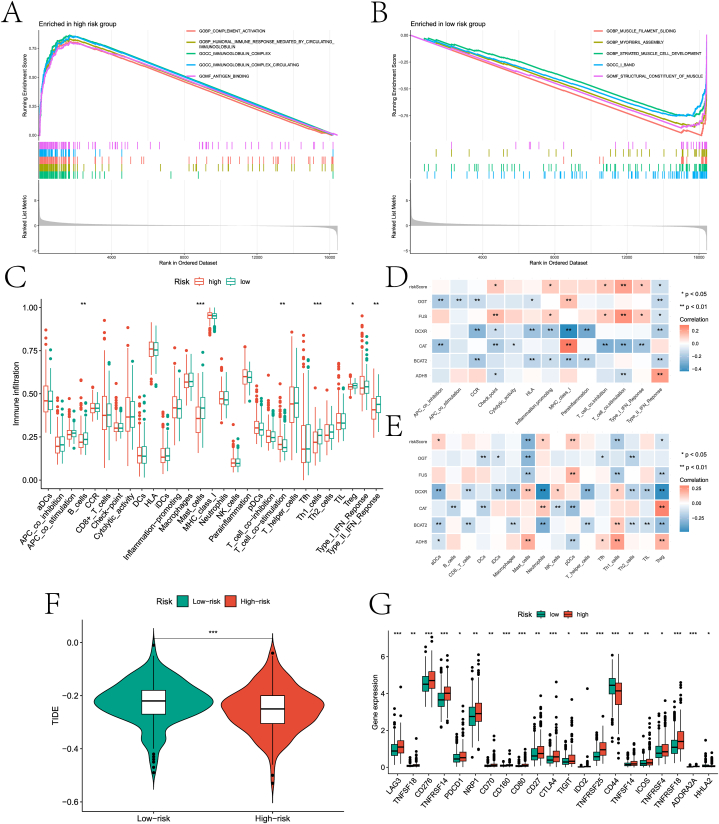
Immune landscape of high and low risk groups. A and B are the first 5 and last 5 pathways of high and low risk groups based on GSEA analysis. C is the difference in the abundance of immune cell infiltration and immune function score between the two groups based on ssGSEA package. D-E is a heat map of correlation between risk scores, prognostic genes, and immune cells/function. F is TIDE analysis for two risk groups. G is the boxplot of the differences in immunoassay sites between risk subgroups.

This paper analyzed the first and last five pathways involved in the high- and low-risk groups based on GSEA (Fig. 11A–B). Multiple immune cell infiltration abundances and multiple immune function scores were evaluated in the high and low-risk groups based on the ssGSEA algorithm (Fig. 11C). Among them, the significance level of mast cells and helper T cells (Th1 cells) was higher (p<0.01). Significant correlations were found between most immune cells, immune function, risk scores, and genes used to construct risk models (Fig. 11D–F). In addition, Tide scores and multiple immune checkpoints significantly differed between high and low-risk groups (Fig. 11G).

## 4 Discussion

In this study, we conducted an in-depth investigation into the development mechanism of prostate cancer (PCa). Through comprehensive analysis of PCa samples and their corresponding controls using RNA-seq and scRNA-seq data, we successfully identified two subtypes of PCa and constructed corresponding prognostic models.

Firstly, this study explored the expression landscape of intersecting genes between differentially expressed genes (DEGs) of various cell clusters from PCa patient and control groups using scRNA-seq data. The expression of intersecting genes identified B cell clusters as highly scored cell clusters. Enriched pathways of DEGs in this cluster, compared with other cell clusters, are mostly associated with the occurrence or treatment of PCa. Upregulation of MHC class I molecules enhanced recognition of mouse PCa cells [14]. Furthermore, we found that the macrophage migration inhibitory factor (MIF) pathway plays a crucial role in communicating B cell populations with others. Mohammad Reza Razzaghi et al. found that MIF-173 polymorphism may be associated with a higher incidence of PCa [15]. In addition, studies have revealed that the activation status of B cells may be associated with the frequency of PCa cell generation, which could contribute to the clinical treatment of PCa. Specifically, the authors investigated the infiltration of B cells in prostate cancer tissues and compared the activation status of B cells and the frequency of cytokine-producing cells in localized and advanced prostate cancer. Significant differences in the activation status of B cells and the frequency of cytokine-producing cells were observed in both scenarios [16].

Second, in order to screen MRGs associated with the prognosis of PCa, differential analysis was conducted based on RNA-seq data from PCa samples and their control group, and the intersection genes were further intersected with previously identified DEGs. Based on univariate Cox regression analysis, 16 prognosis-related genes were screened from the intersection genes. Some genes have been confirmed to be closely related to the development of PCa. Ramesh E Ghanbarpanah et al. found that FUS plays a key role in androgen receptor signaling and cell cycle progression in PCa [17]. Therefore, exogenous expression of FUS may be a potential therapeutic approach for PCa. Genomic instability of BNIP3L may contribute to more aggressive PCa in black males [18]. showed that PABPC1 is a novel co-regulator of androgen receptors and may be a potential target to block androgen receptor activation in castration-resistant PCa [19]. Liu et al. identified CDK5RAP3 as one of the genes for constructing a PCa risk model through a comprehensive analysis of cell cycle-related genes in PCa patients. Zhang et al. also identified ADH5 as a prognosis-related gene by bioinformatics analysis [20]. Studies by Ninu Poulose et al. have shown that VPRBP/DCAF1 promotes prostate cancer cell proliferation by inhibiting p53 activation under the influence of AR and OGT [21]. Furthermore, MYO6 is strongly associated with Gleason score in prostate cancer [22].

Thirdly, in order to achieve effective stratification of PCa patients, this study identifies PCa subtypes based on the NMF algorithm and prognostic genes. Patients with two subtypes exhibit significant differences in survival, immune cell infiltration abundance/immune function scores. Meng et al. determined that B cells were highly enriched in PCa samples by NMF algorithm [23]. Heidi Hempel Sullivan et al. found that extra-PCa mast cells lead to higher cancer invasion based on a wild-type mouse model [24]. Diane L Costanzo-Garvey et al. found that neutrophils are mediators of metastatic PCa progression in bone [25]. NK cells isolated from peripheral blood of patients acquire properties associated with pro-inflammatory angiogenesis of endothelial cells, recruit monocytes and polarize macrophages to an M2-like phenotype [26]. Erasmia T Xanthopoulou et al. found radiation-induced activation of type I interferon-related pathways in PCa cell lines [27]. Additionally, through GSVA analysis of two subtypes of samples, multiple pathways showing significant differences between the two groups have been confirmed to be associated with the prognosis/treatment of prostate cancer. For instance, the bone-specific m6a modification of eRNA has been demonstrated to play a crucial role in regulating PCa progression and radiotherapy resistance [28]. Zhang et al. designed and synthesized a series of novel l-phenylalanine dipeptides. Among them, compound 7c exhibited potent anti-tumor activity both in vitro and in vivo by inducing apoptosis in PCa cells PC3 [29]. Tyrosine kinase inhibitors (TKIs) as a therapeutic approach for various malignant tumors have been extensively studied. Michelle A Ojemuyiwa et al. found that using these treatment methods could influence the bone microenvironment and reduce the incidence of PCa-related morbidity [30].

Fourth, to guide stratified treatment of clinical PCa patients, a reliable prognostic model was constructed based on Lasso-Cox regression analysis in this study. Further validation with TCGA and GEO test sets confirmed that the model can effectively stratify PCa patients into two groups with significantly different survival outcomes. Through the GSEA analysis of the high and low-risk groups, this paper identified the Top pathways of the two groups. Most of these pathways have been shown to play essential roles in PCa progression. Giovanni Stallone et al. demonstrated the regulatory effect pentraxin-3 on PCa complement activation [31]. Beneduce et al. speculated that PSA-IgM might be a complementary serological marker of PCa by gaining in cancer detection using a combination of prostate-specific antigen (PSA) and prostate-specific antigen-immunoglobulin M (PSA-IgM) [32]. Studies by Berna Uygur et al. showed that interactions with muscle cells promote PCa cell fusion, stemness, and drug resistance [33].

Finally, to explore the difference in the abundance of immune cell infiltration in high-risk and low-risk samples, this paper identified the immune cells (mast cells and helper T cells (Th1 cells)) with significant differences in infiltration abundance in the two groups based on the ssGSEA algorithm. This is consistent with the significantly different cells identified by subtype. In addition, the expression of multiple immune checkpoint loci (LAG3, CD276, TNFRSF14, CD160, CD80, CTLA4, ICO2, TNFRSF25, CD44, TNFRSF18, and ADORA2A) was found to be significantly different between the two groups (p<0.01). CD276 is highly expressed in PCa and associated with early recurrence and metastasis [34]. The differential expression of CD160 and CD80 in the two groups was consistent with the bioinformatics analysis results of Feng et al. Anti-PD-1-CTLA4 has a positive effect in combinatorially combating PCa [35]. Hao et al. reviewed CD44 as a potential therapeutic target for metastatic PCa [36].

The research presented in this paper has certain limitations. Firstly, the study may be constrained by the number and sources of samples. Although comprehensive analysis was conducted using RNA-seq and scRNA-seq data, potential issues such as insufficient sample size or sample source imbalance may affect the reliability and generalizability of the results. Secondly, the study relies on bioinformatics analysis tools and techniques such as the R packages “Seurat” and “limma”. The analysis results may be influenced by factors such as data processing methods and parameter selection, potentially introducing limitations and biases. Thirdly, despite validation of the model on internal and external test sets, there may still be biases in sample selection for validation and consistency issues with data sources. Additionally, the predictive capability of the model may be influenced by unaccounted variables or factors. Lastly, while the study proposes a risk model and prognostic genes as references for developing therapeutic targets for prostate cancer, the generalizability of the results may be influenced by other factors such as different populations, environments, and treatment regimens.

## 5 Conclusion

Here, we identified subtypes of PCa and constructed a risk model by integrating scRNA-seq and RNA-seq. We observed differences in prognosis and immunity among different subtypes and different risk groups. For example, high-risk groups are associated with poorer survival outcomes. In conclusion, this paper found that mitophagy is closely related to PCa. MRGs provide new genetic markers for predicting the prognosis of PCa patients.

## Funding

This work was supported by the 10.13039/501100010251Youth Science Foundation of Jiangxi Province, Education Department of Jiangxi Province, China (GJJ211550 to Ruo-Hui Huang); the General Project from the Health Commission of Jiangxi Province, China (202310780 to Ruo-Hui Huang).

## Data availability statement

The data of the paper were downloaded from the TCGA database (https://portal.gdc.cancer.gov/) and the GEO database (https://ncbi.nlm.nih.gov/geo/). The datasets from the GEO database are numbered GSE153892 and GSE70770.

## CRediT authorship contribution statement

**Zong-Yan Liu:** Writing – original draft, Visualization, Validation, Software, Methodology, Formal analysis. **Ruo-Hui Huang:** Writing – review & editing, Supervision, Software, Formal analysis, Conceptualization.

## Declaration of competing interest

The authors declare the following financial interests/personal relationships which may be considered as potential competing interests: Ruohui Huang reports was provided by First Affiliated Hospital of Gannan Medical University. If there are other authors, they declare that they have no known competing financial interests or personal relationships that could have appeared to influence the work reported in this paper.
