# Supplementary material for: Integrating single-cell RNA-sequencing and bulk RNA-sequencing data to explore the role of mitophagy-related genes in prostate cancer
Source: Heliyon. 2024 May 9;10(9):e30766. doi: 10.1016/j.heliyon.2024.e30766 (PMC11107114; doi:10.1016/j.heliyon.2024.e30766)
Supplement: Multimedia component 1 [file mmc1.docx]

# Supplementary Materials

Table S1 Baseline data of TCGA data

| Characteristics | High-risk | Low-risk | P value |
| --- | --- | --- | --- |
| n | 198 | 198 |  |
| Age, mean ± sd | 61.798 ± 6.2867 | 59.985 ± 7.1535 | 0.008 |
| T, n (%) |  |  | 0.001 |
| T2 | 57 (14.6%) | 92 (23.5%) |  |
| T3 | 135 (34.5%) | 100 (25.6%) |  |
| T4 | 4 (1%) | 3 (0.8%) |  |
| N, n (%) |  |  | < 0.001 |
| N0 | 136 (39.5%) | 148 (43%) |  |
| N1 | 48 (14%) | 12 (3.5%) |  |

n represents the number of samples, while T and M represent the T and M stages of the tumor, respectively.


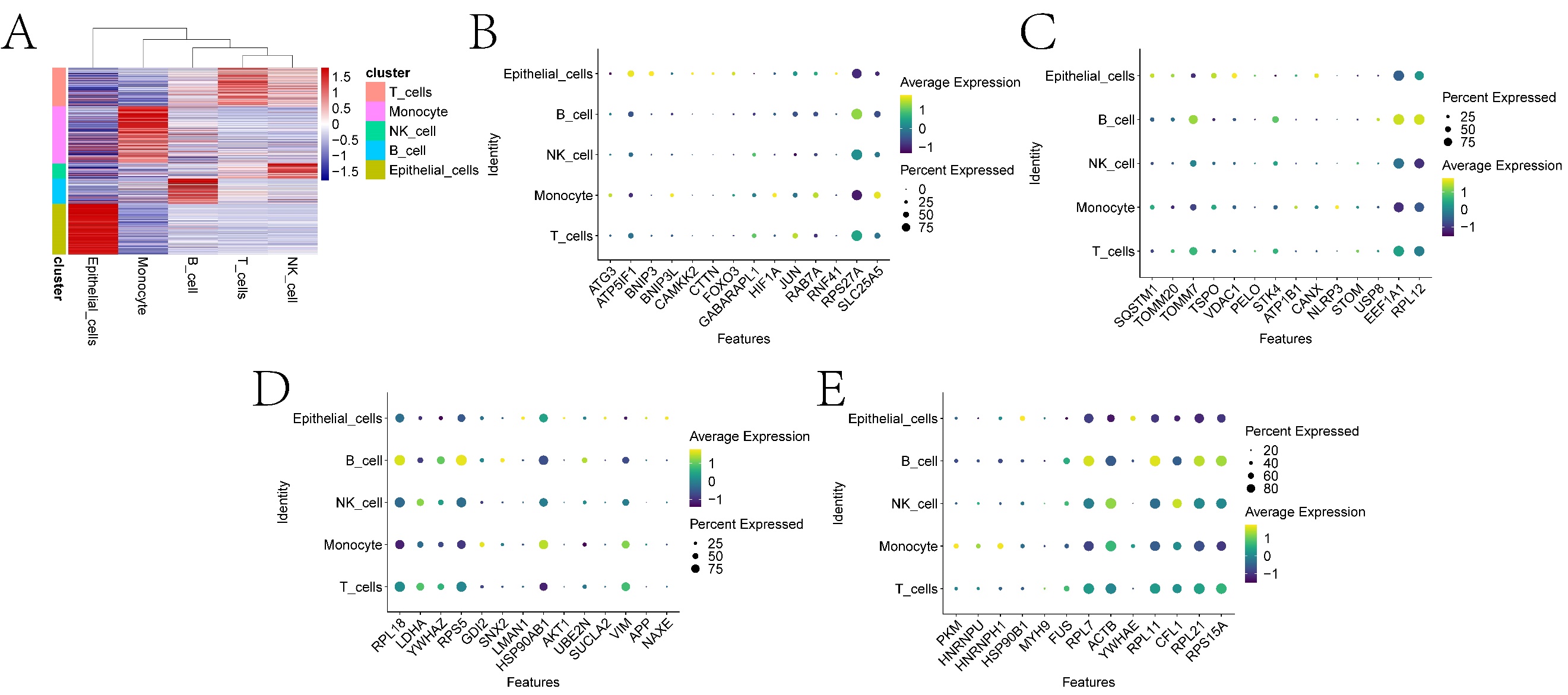


Figure S1 Analysis of differences among cell populations and expression of intersection genes. A is the heat map of DEGs expression in each cell population. B-E shows the expression of intersection genes between different cell populations.


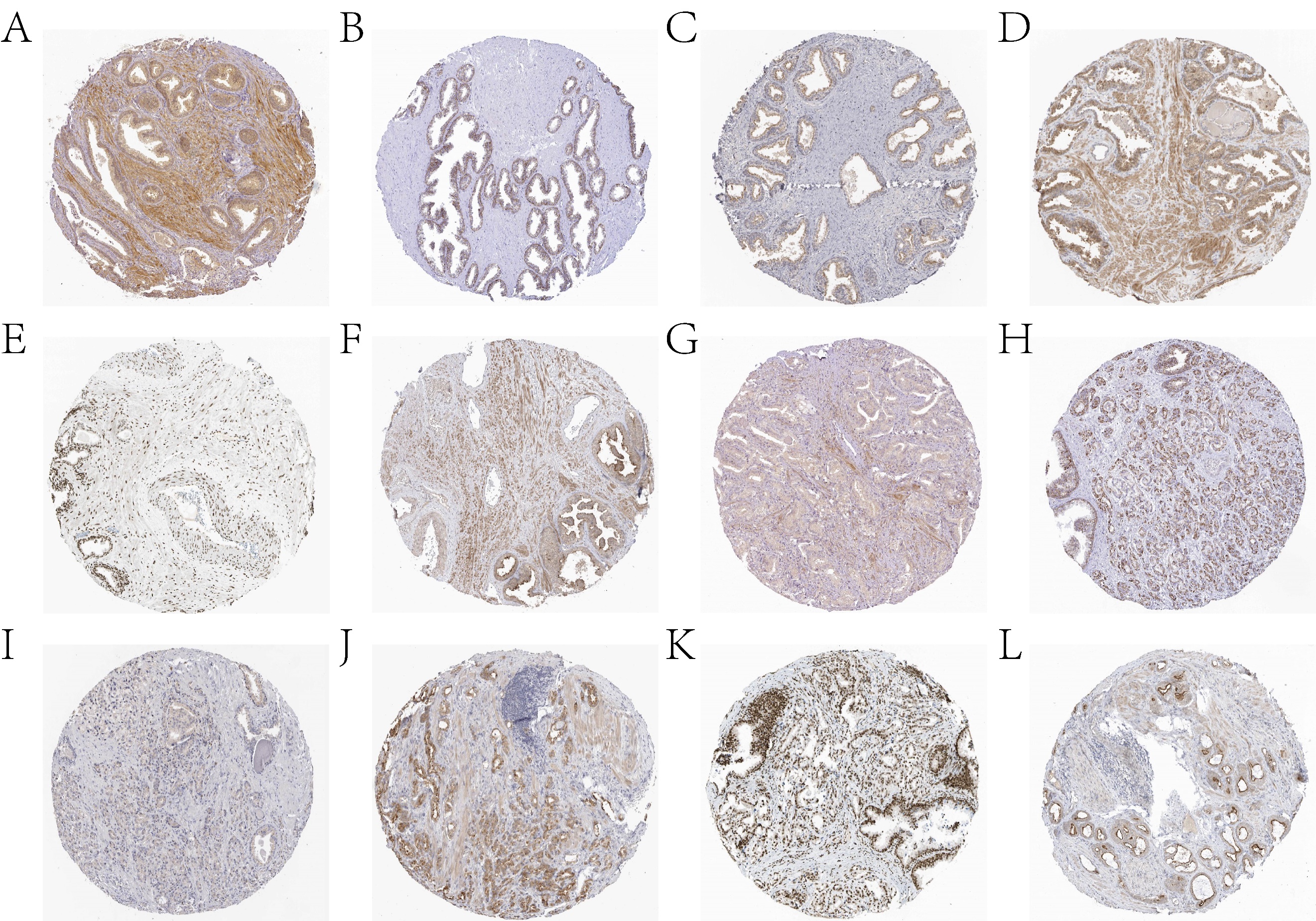


Figure S2 Immunohistochemical staining showed the expression of six genes at the protein level. A-F are immunohistochemical staining images of ADH5, BCAT2, CAT, DCXR, FUS and OGT in normal tissues, respectively. G-L are the immunohistochemical staining images of ADH5, BCAT2, CAT, DCXR, FUS and OGT in PCa tissues, respectively.

Table S2 A list of MRGs

| MRGs |
| --- |
| AMBRA1 |
| ARFIP2 |
| ATF4 |
| ATG13 |
| ATG14 |
| ATG2A |
| ATG2B |
| ATG3 |
| ATG4B |
| ATG4D |
| ATG5 |
| ATG7 |
| ATG9A |
| ATG9B |
| ATP13A2 |
| ATP5IF1 |
| BCL2L1 |
| BECN |
| BNIP3 |
| BNIP3L |
| CAMKK2 |
| CDC37 |
| CERS1 |
| CISD2 |
| CSNK2A |
| CSNK2A1 |
| CSNK2A2 |
| CSNK2B |
| CTSK |
| CTTN |
| DNM1L |
| EIF2AK3 |
| FBXL4 |
| FBXW7 |
| FIS1 |
| FOXO3 |
| FUNDC1 |
| FUNDC2 |
| FZD5 |
| GABARAP |
| GABARAPL1 |
| GABARAPL2 |
| GABARAPL3 |
| GBA |
| GSK3A |
| HAX1 |
| HDAC6 |
| HIF1A |
| HK2 |
| HRAS |
| HTRA2 |
| HTT |
| HUWE1 |
| JNK |
| JUN |
| LRBA |
| MAP1LC |
| MAP1LC3A |
| MAP1LC3B |
| MAP1LC3B2 |
| MAP1LC3C |
| MARK2 |
| MFN1 |
| MFN2 |
| MITF |
| MRAS |
| MTERF3 |
| MUL1 |
| NRAS |
| OGT |
| PARK2 |
| PARK7 |
| PARL |
| PGAM5 |
| PHB2 |
| PINK1 |
| PRKN |
| RAB7A |
| RAB7B |
| RB1CC1 |
| RELA |
| RETREG1 |
| RHOT1 |
| RHOT2 |
| RIMOC1 |
| RNF41 |
| RP-L40e |
| RPS27A |
| RP-S27Ae |
| RRAS |
| RRAS2 |
| SLC25A4 |
| SLC25A46 |
| SLC25A5 |
| SNX30 |
| SNX7 |
| SP1 |
| SPATA18 |
| SPATA33 |
| SQSTM1 |
| SRC |
| SREBF1 |
| SREBF2 |
| TAFAZZIN |
| TFE3 |
| TIGAR |
| TOMM20 |
| TOMM22 |
| TOMM40 |
| TOMM6 |
| TOMM7 |
| TOMM70 |
| TP53 |
| TSC2 |
| TSPO |
| UBA52 |
| UBB |
| UBC |
| ULK1 |
| ULK2 |
| USP36 |
| VDAC1 |
| VPS13C |
| VPS13D |
| WDR45 |
| WDR45B |
| WIPI1 |
| WIPI2 |
| SOD2-OT1 |
| CLEC16A |
| ABCE1 |
| PHB1 |
| FKBP8 |
| TRIM27 |
| MIR7-3HG |
| VDAC2 |
| CHUK |
| CNOT4 |
| EMSLR |
| OPA1 |
| CALCOCO2 |
| BDNF-AS |
| PELO |
| H19 |
| CERNA3 |
| MTX1 |
| PRKAA1 |
| ATG4A |
| LRRK2 |
| ATAD3A |
| MTARC2 |
| PTRH2 |
| VCP |
| BCL2L13 |
| SNCA |
| LINC01554 |
| MAP2K2 |
| WDR26 |
| MON1A |
| SAMM50 |
| LINC01672 |
| MDH1 |
| VDAC3 |
| IMMT |
| MAPK14 |
| BCAS3 |
| PHAF1 |
| LINC-PINT |
| STK4 |
| TDRKH |
| GBA1 |
| FANCC |
| LRPPRC |
| MFF |
| TUFM |
| PI4KB |
| PDK1 |
| ATP1B1 |
| HSPA8 |
| CANX |
| CHCHD3 |
| NBR1 |
| TRAP1 |
| SMAD5-AS1 |
| SESN2 |
| TMX2-CTNND1 |
| MTX2 |
| MRPS2 |
| PEX13 |
| TRA-TGC7-1 |
| TRA-TGC5-1 |
| SCARNA5 |
| KRT15 |
| NLRP3 |
| UBXN6 |
| RNU6-1 |
| RNU6-2 |
| RNU6-7 |
| RNU6-8 |
| RNU6-9 |
| RNU6-1-001 |
| RNU6-1-002 |
| RNU6-1-003 |
| RNU6-1-004 |
| TBC1D5 |
| STX10 |
| NIPSNAP1 |
| PI4K2A |
| STOM |
| MARCHF5 |
| ALB |
| BCL2 |
| USP8 |
| UQCRC2 |
| MIEF1 |
| EEF1A1 |
| RPL12 |
| TBC1D15 |
| RPL18 |
| LDHA |
| YWHAZ |
| MTPAP |
| IRGM |
| GLS |
| PIP4K2C |
| RPS5 |
| ABCD3 |
| CYP51A1 |
| STT3B |
| GDI2 |
| SNX2 |
| PGR-AS1 |
| MAVS |
| ATG16L1 |
| LMAN1 |
| ACIN1 |
| NUP93 |
| CRNKL1 |
| HSPA9 |
| NIPSNAP2 |
| HSP90AB1 |
| AKT1 |
| STOML2 |
| PFKP |
| CHAF1B |
| FXR2 |
| RNF31 |
| BAG6 |
| MAPK9 |
| TFRC |
| CLINT1 |
| SRSF4 |
| UQCRC1 |
| MAP2K7 |
| NAA10 |
| NAA16 |
| IDH2 |
| HK1 |
| UBE2N |
| NDUFV1 |
| UBXN1 |
| MEG3 |
| LONP1 |
| SUCLA2 |
| MRPL37 |
| OCIAD1 |
| SIAH3 |
| TIMM44 |
| BLOC1S1 |
| PRPF8 |
| VIM |
| DSP |
| FADS2 |
| SLC25A24 |
| TOM1 |
| MON2 |
| GDAP1 |
| APP |
| NAXE |
| SCAMP3 |
| PRKCD |
| HNRNPM |
| ATAD3B |
| MAPK8 |
| PRKAA2 |
| CCZ1B |
| PKM |
| TUBB4B |
| HNRNPU |
| HNRNPH1 |
| TIMM23 |
| MAP2K3 |
| EMC1 |
| GAPDH |
| HSP90B1 |
| MYH9 |
| FUS |
| CCT3 |
| RPL7 |
| NPLOC4 |
| ARIH1 |
| ACTB |
| FASN |
| YWHAE |
| RPL11 |
| CFL1 |
| MCM7 |
| SPTBN1 |
| TRIM25 |
| EPRS1 |
| PLEC |
| RPL21 |
| RPS15A |
